# Antiparasitic activity of FLLL-32 against four *Babesia* species, *B. bovis*, *B. bigemina*, *B. divergens* and *B. caballi*, and one *Theileria* species, *Theileria equi in vitro*, and *Babesia microti* in mice

**DOI:** 10.3389/fphar.2023.1278451

**Published:** 2023-11-02

**Authors:** Shimaa Abd El-Salam El-Sayed, El-Sayed El-Alfy, Hanadi B. Baghdadi, Mohamed Z. Sayed-Ahmed, Saad S. Alqahtani, Nawazish Alam, Sarfaraz Ahmad, Md. Sajid Ali, Ikuo Igarashi, Mohamed Abdo Rizk

**Affiliations:** ^1^ National Research Center for Protozoan Diseases, Obihiro University of Agriculture and Veterinary Medicine, Obihiro, Japan; ^2^ Department of Biochemistry and Chemistry of Nutrition, Faculty of Veterinary Medicine, Mansoura University, Mansoura, Egypt; ^3^ Parasitology Department, Faculty of Veterinary Medicine, Mansoura University, Mansoura, Egypt; ^4^ Biology Department, College of Science, Imam Abdulrahman Bin Faisal University, Dammam, Saudi Arabia; ^5^ Basic and Applied Scientific Research Center (BASRC), Imam Abdulrahman Bin Faisal University, Dammam, Saudi Arabia; ^6^ Department of Clinical Pharmacy, College of Pharmacy, Jazan University, Jizan, Saudi Arabia; ^7^ Department of Clinical Pharmacy, College of Pharmacy, King Khalid University, Abha, Saudi Arabia; ^8^ Department of Pharmaceutics, College of Pharmacy, Jazan University, Jizan, Saudi Arabia; ^9^ Department of Internal Medicine and Infectious Diseases, Faculty of Veterinary Medicine, Mansoura University, Mansoura, Egypt

**Keywords:** FLLL-32, *Babesia*, *Theileria equi*, *in vitro*, *in vivo*

## Abstract

**Introduction:** FLLL-32, a synthetic analog of curcumin, is a potent inhibitor of STAT3’s constitutive activation in a variety of cancer cells, and its anticancer properties have been demonstrated both *in vitro* and *in vivo*. It is also suggested that it might have other pharmacological activities including activity against different parasites.

**Aim:** This study therefore investigated the *in vitro* antiparasitic activity of FLLL-32 against four pathogenic *Babesia* species, *B. bovis*, *B. bigemina*, *B. divergens*, and *B. caballi*, and one *Theileria* species, *Theileria equi*. *In vivo* anti-Babesia microti activity of FLLL-32 was also evaluated in mice.

**Methods:** The FLLL-32, in the growth inhibition assay with a concentration range (0.005–50 μM), was tested for it’s activity against these pathogens. The reverse transcription PCR (RT-PCR) assay was used to evaluate the possible effects of FLLL-32 treatment on the mRNA transcription of the target *B. bovis* genes including *S-adenosylhomocysteine hydrolase* and *histone deacetylase*.

**Results:** The *in vitro* growth of *B. bovis*, *B. bigemina*, *B. divergens*, *B. caballi*, and *T. equi* was significantly inhibited in a dose-dependent manner (in all cases, *p* < 0.05). FLLL-32 exhibits the highest inhibitory effects on *B. bovis* growth *in vitro*, and it’s IC_50_ value against this species was 9.57 μM. The RT-PCR results showed that FLLL-32 inhibited the transcription of the *B. bovis S-adenosylhomocysteine hydrolase* gene. *In vivo*, the FLLL-32 showed significant inhibition (*p* < 0.05) of *B. microti* parasitemia in infected mice with results comparable to that of diminazene aceturate. Parasitemia level in *B. microti*-infected mice treated with FLLL-32 from day 12 post infection (pi) was reduced to reach zero level at day 16 pi when compared to the infected non-treated mice.

**Conclusion:** The present study demonstrated the antibabesial properties of FLLL-32 and suggested it’s usage in the treatment of babesiosis especially when utilized in combination therapy with other antibabesial drugs.

## 1 Introduction

The most often used babesiacides for treating babesiosis in animals are diminazene aceturate (DA) and imidocarb dipropionate (ID) (Vial and Gorenflot, 2006). Studies have revealed that ID can remain in the products of treated animals for a long time after stopping the treatment (Mosqueda et al., 2012). Therefore, there is a paucity of it’s supply in some nations (Vial and Gorenflot, 2006; Mosqueda et al., 2012). Because of the effectiveness of multiple *in vitro* and *in vivo* culture methods, it is possible to used them to discover novel, and effective anti-piroplasmid compounds (Keroack et al., 2019). In this regard, several compounds recently demonstrated anti-piroplasm effects both *in vitro* and *in vivo*. For instance, the Malaria Box was used to screen for new potent antipiroplasm medications, such as 3-[(2-Hydroxyethyl)amino]-5-methylphenazin-5-ium “MMV396693,” by testing it against the growth of several bovine *Babesia* and equine piroplasms *in vitro* and the *B. microti* parasite *in vivo* (Rizk et al., 2019; Rizk et al., 2022). The mode of action for the antibabesial efficacy of MMV396693 is still unknown. However, our previous study (Rizk et al., 2022) suggested that both MMV396693 and ID might have a similar mode of action for inhibition *Babesia* parasite. The effectiveness of such compounds in combination therapy with other medication candidates can therefore be tested. In this concern, FLLL-32 is a synthetic analog of curcumin, which replaces the two hydrogens on the middle carbon with spiro-cycloalkyl rings to generate a diketo form. Such a chemical modification can prevent the enolization of FLLL32, thereby making it more stable (Lin et al., 2010b). Moreover, FLLL-32 can overcome limitations associated with using curcumin itself as poor bioavailability, low aqueous solubility, and rapid metabolism (Esatbeyoglu et al., 2012; Stanić, 2017; Morshedi et al., 2021).

FLLL-32 attracted a lot of attention due to its anti-infective, anti-mutagenic, anti-cancer, natural antioxidant, antimicrobial, and anti-inflammatory properties (Hussain et al., 2017; Huang et al., 2022). In addition, it specifically inhibits STAT3 and retains the cellular response to cytokines with anti-tumor activity on different types of cancer cells including colorectal cancer, glioblastoma, multiple myeloma, and liver cancer cells (Lin et al., 2010a). Despite the wide use of FLLL-32 as an anticancer, its antiprotozoal activity has not been determined yet. For that, the objective of this study was to evaluate the *in vitro* antiprotozoal activity of FLLL-32 against *B. bovis*, *B. bigemina*, *B. divergens*, *B. caballi*, and *T. equi* and to assess it’s possible synergistic interaction with two antibabesial drugs, DA and ID, and MMV396693 against these species. Additionally, its *in vivo* inhibitory efficacy on *B. microti* growth in mice was assessed. Eventually, this study also aimed for to provide a preliminary investigation of the underlying molecular mechanism of FLLL-32 action.

## 2 Materials and methods

### 2.1 Chemicals

The compound FLLL-32 was obtained from (Merck KGaA 64271 Darmstadt, Germany) and dissolved in (0.1%) dimethyl sulfoxide (DMSO) for stock concentration and kept at −30°C until use. The antibabesial drug, DA (Ganaseg, Ciba-Geigy Japan Ltd., Tokyo, Japan) was employed as a control drug. DA, ID (Sigma-Aldrich, Tokyo, Japan), and MMV396693 (MolPort, Latvia) were used for the *in vitro* combination inhibition assay. The nucleic acid stain SYBR Green I (SGI) (Lonza, Rockland, United States; 10,000x) was stored at −20°C and thawed before use. A lysis solution comprising Tris (130 mM; pH 7.5), EDTA (10 mM), saponin (0.016%; W/V), and TritonX-100 (1.6%; V/V) had been prepared in advance and stored at 4°C. Both SGI and lysis buffer were used for inhibition assay either *in vitro* or *in vivo*.

### 2.2 Maintenance of the parasites *in vitro*

Using a microaerophilic stationary-phase culture technique, *B. bovis* (Texas strain), *B. bigemina* (Argentina strain), *B. divergens* (German strain), *B. Caballi* (USDA strain), and *Theileria equi* (USDA strain) were grown and maintained in purified bovine or equine red blood cells (RBCs). *Babesia caballi* was cultured in RPMI 1640 medium, whereas *B. bovis*, *B. bigemina*, and *T. equi* were cultured in Medium 199 (both media were purchased from Sigma-Aldrich). For equine *Babesia* and *Theileria* parasites, 40% normal horse serum was added to the media, whereas media was supplemented with 40% normal bovine serum for bovine *Babesia* parasites, along with penicillin G at 60 units per mL, streptomycin at 60 mg/mL, and amphotericin B at 0.15 mg/mL (all from Sigma-Aldrich). *Theileria equi* cultures were supplied with 13.6 g of hypoxanthine (ICN Biomedicals, Inc., United States) per mL. All parasite cultures were grown at 37°C in a 5% CO_2_, 5% O_2_, and 90% N_2_ environment.

### 2.3 *In vitro* growth inhibition assay and viability test

The fluorescence assay using an SGI stain was employed to study the effect of FLLL-32 on *Babesia*/*Theileria* growth (Rizk et al., 2015; Rizk et al., 2016). Double 96-well plates (Nunc, Roskilde, Denmark) were utilized to culture bovine *Babesia* as well as equine *Babesia* and *Theileria* in the infected pRBCs with either media only (blank wells) or a medium containing 0.005–200 μM FLLL-32. Positive control cultures, on the other hand, were treated with DA concentration ranging from 0.25 to 10 μM. Negative experimental controls included wells containing only the pRBCs with media containing the used solvent (0.1% DMSO). The plates were then incubated for 4 days at 37°C, and the IC_50_ values for FLLL-32 and DA were calculated on the 4th day based on growth inhibition in three separate experiments. On the fourth day of treatment, the viability assay was carried out by mixing 1.5 μL of the control or FLLL32-treated infected RBCs with 3.5 μL of parasite-free RBCs, suspending the mixture in fresh growth medium without the addition of drugs, and incubating the mixture at 37°C for the following 4 days without changing the medium (Rizk et al., 2016). The IC_50_ values of FLLL32 were calculated using the non-linear regression curve fit in GraphPad Prism 5.0 (GraphPad Software, Inc., San Diego, CA, United States) (Rizk et al., 2016; Rizk et al., 2017; Rizk et al., 2019; Rizk et al., 2021a; Rizk et al., 2021b; El-Sayed et al., 2023).

Combination therapies of FLLL-32 with the commonly used (DA, and ID), and the recently identified antibabesial drugs (MMV396693) were evaluated. The combination ratios ranged from 0.50 to 0.75 IC_50_s of the selected drugs (Supplementary Table S1) were prepared as previously described (Rizk et al., 2023). A 96-well plate containing *B. bovis*, *B. bigemina*, *B. caballi*, and *T. equi* pRBCs was treated with a two-drug combination; FLLL-32+DA, FLLL-32+ID, and FLLL-32+MMV396693 at concentrations of 0.5 x IC_50_ and 0.75 x IC_50_ in triplicates.

All *in vitro* tests were conducted at 1% parasitemia, and either 2.5% hematocrit (HCT) for *B. bigemina* and *B. bovis*, or 5% HCT for *B. divergens*, *B. caballi*, and *T. equi* parasites (Rizk et al., 2017). Next, 100 μL lysis buffer mixed with a 2× SGI was added to each well in the 96-wells plates after 4 days of incubation. The mean fluorescence values were then plotted against the logarithm of drug concentrations. Each drug concentration was tested in triplicate in each experiment and values of fluorescence assays were calculated from three separate experiments. The parasite survival was evaluated using non-linear expression analyses (Schadich et al., 2022).

### 2.4 Reverse transcription-PCR

Reverse transcription-PCR (RT-PCR) was used to assess the effect of FLLL-32 treatment on the mRNA transcription of the target *B. bovis* genes including *S-adenosylhomocysteine hydrolase* (*BbSAHH*) and the *Histone deacetylase* (*BbHDAC3*) (El-Sayed et al., 2023). *Babesia bovis* was grown in bovine RBCs on 24-well culture plates (Nunc, Roskilde) as previously described and was treated for 8 h with FLLL-32 at the 99% inhibitory concentration (IC99) (18.94 μM) and 0.1% DMSO as a negative control. After that, pRBCs were collected and washed with phosphate buffer saline. Following the manufacturer’s guidelines, total RNA was extracted using a commercial RNeasy mini kit (QIAGEN, Germantown Rd, Germantown, MD, United States). A Nanodrop 2000 spectrophotometer (Thermo Fisher Scientific Inc., Tokyo, Japan) was used to measure the RNA concentration, and one Step RNA Kit (AMV) (Takara, Japan) was used for conducting the RT-PCR following the manufacturer’s instructions. The *B. bovis profilin* (*BbPROF*) gene is used as the reference control gene. The *BbSAHH*, *BbHDAC3*, and *BbPROF* genes were amplified using total RNA (150 ng) from the treatment cultures and the control. The specific forward and reverse primers used are listed in Table 1, and the PCR conditions were 30 min at 50°C for reverse transcription, followed by 2 min of 94°C denaturation, 30 cycles of 94°C for 30 s denaturation, 55°C for 30 s annealing for *BbHDAC3*, *BbPROF*, and 57°C for *BbSAHH*, 1 min of 72°C extension, and 5 min of 72°C final extension (Mukhjargal et al., 2012). After staining with ethidium bromide, the amplified products were electrophoresed on 2.0% (w/v) agarose gels and visualized with a UV transilluminator (Nippon Gene, Tokyo, Japan). Gel electrophoresis bands were analyzed using ImageJ software (Schadich et al., 2012; El-Sayed et al., 2023).

**TABLE 1:** Gene-specific primers for amplifying *BbSAAH*, *BbHDAC3*, and *BbPROF* genes.

Gene^a^	Gene oligonucleotide primer	References
*BbSAAH*	F 5′ - CAT​GCA​CTG​GTG​GTA​TCG​AC-3′	This study
	R 5′ - AGC​CGG​GTT​TGA​TGT​TAG​TG-3′	
*BbHDAC3*	F 5′ - ACG​AAT​TCA​TGG​AGA​AGA​GAG​TTT​CTT​A -3′	Mukhjargal et al. (2012)
	R 5′ - ACC​TCG​AGC​TAT​ATC​GGT​ATA​TGC​TGG​T -3′	
*BbPROF*	F 5′ - ACG​AAT​TCA​TGG​CAG​ATT​GGG​TTC-3′	El-Sayed et al. (2023)
	R 5′ -ACC​TCG​AGT​TAA​TAA​CCA​TTG​GCA​GCC-3′	

^a^ *Babesia bovis S-adenosylhomocysteine hydrolase* (*BbSAHH*), *B. bovis Histone deacetylase* (*BbHDAC3*), and *B. bovis profilin* (*BbPROF*).

### 2.5 *In vivo* efficacy of FLLL-32 on the growth of *B. microti* in infected mice

Four groups of BALB/c mice (*n* = 5 per group) aged 8 weeks (CLEA, Tokyo, Japan) were injected intraperitoneally with 1 × 10^7^ *B. microti* (Munich strain) iRBCs except for the mice in the first group which remained uninfected and served as a negative control. When infected mice demonstrated 1% parasitemia, mice in two experimental groups were given daily injections of tested drugs (FLLL-32 and DA) for 5 days whereas, one group was non-treated and served as a control group. One group was treated with FLLL-32 intraperitoneal at a dosage of 50 mg/kg while, the other group was given DA intraperitoneal at a dosage of 25 mg/kg (positive control). A venous tail blood sample (2.5 μL) was collected from each mouse and transferred to a 96-well plate with RPMI 1640 Medium previously mixed with 50 μl of lysis solution. Following that, 50 μL of lysis buffer with 2x SGI nucleic acid stain was mixed into each well. Eventually, the plate was incubated in the dark for 1 h. The inhibitory effects of FLLL-32 and DA on the growth of *B. microti* were evaluated using a fluorescence spectrophotometer every 48 h until 30 days post-inoculation. Following the completion of the study, all of the mice were euthanized humanely via inhalation of the chemical chloroform, which was followed by neck dislocation (physical euthanasia).

### 2.7 Statistical analysis

The obtained data were analyzed using GraphPad Prism. Differences between the control and treated groups were determined by one-way analysis of variance (ANOVA) and unpaired *t*-tests. The statistical significance was defined as *p*-value < 0.05. The statistically significant differences between the drug-treated and positive-control groups were used in the viability test as an indication of parasite regrowth (Rizk et al., 2016).

### 2.8 Ethics approval and consent to participate

All experimental protocols in this work were approved by the Animal Care and Use Committee at Obihiro University of Agriculture and Veterinary Medicine (Approval No. 27-65). All experiments were carried out following the Fundamental Guidelines for the Proper Conduct of Animal Experiment and Related Activities at Academic Research Institutions issued by Japan’s Ministry of Education, Culture, Sports, Science, and Technology. The pathogen experiment’s IDs were as follows: *Babesia microti*: 20170905; equine piroplasm parasites: 201910-2; and bovine *Babesia*: 201708-4.

## 3 Results

### 3.1 FLLL-32 inhibits *B. bovis* growth *in vitro*

**FIGURE 1:**
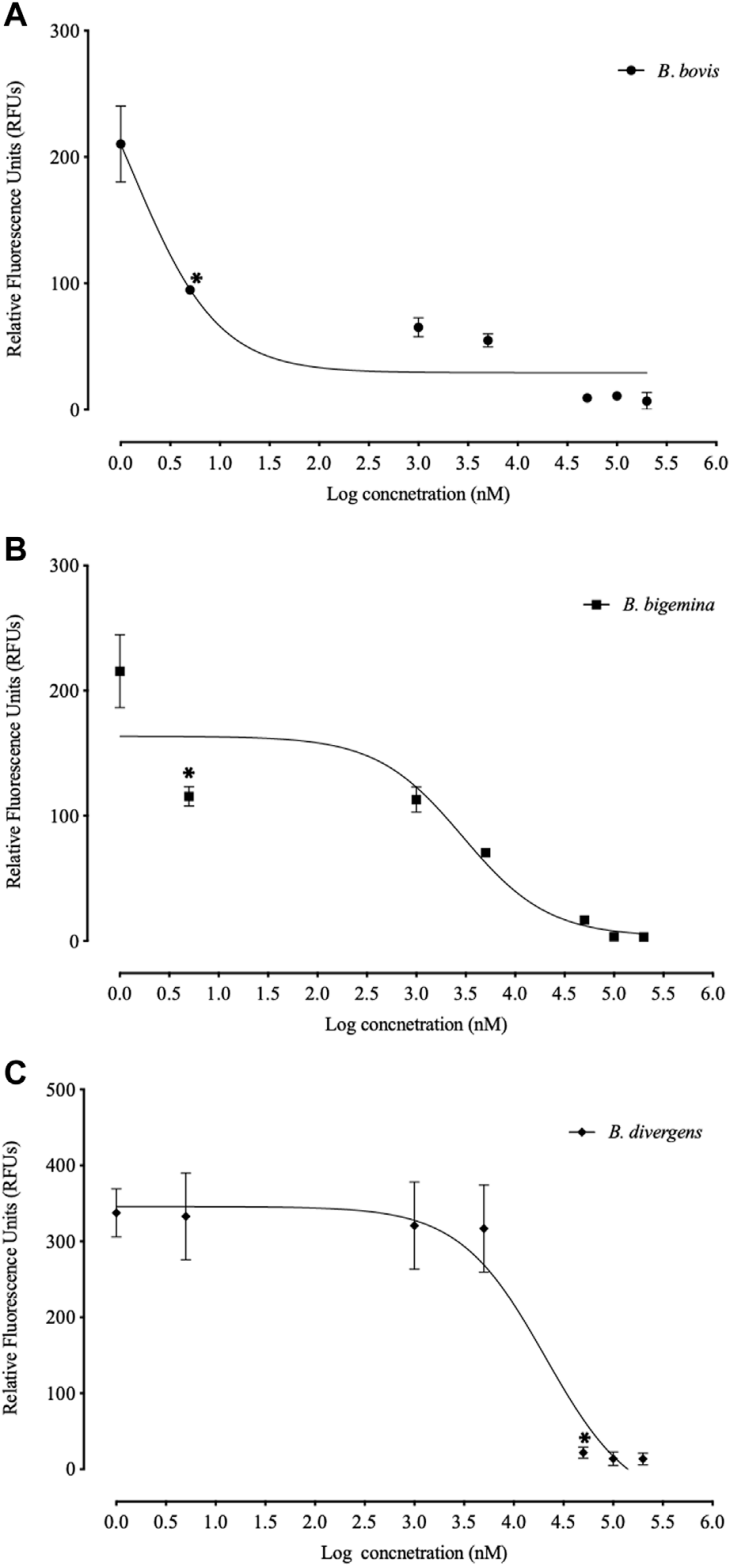
Correlation between relative fluorescence units (RFUs) and the log concentrations of FLLL-32 (µm) on bovine *Babesia* parasites. **(A)** *B. bovis*. **(B)** *B. bigemina*. **(C)** *B. divergens.* Each value represents the mean of triplicate wells after subtraction of the background fluorescence for non-parasitized RBCs. The logarithmic values of drug concentration are of the base 10.

**FIGURE 2:**
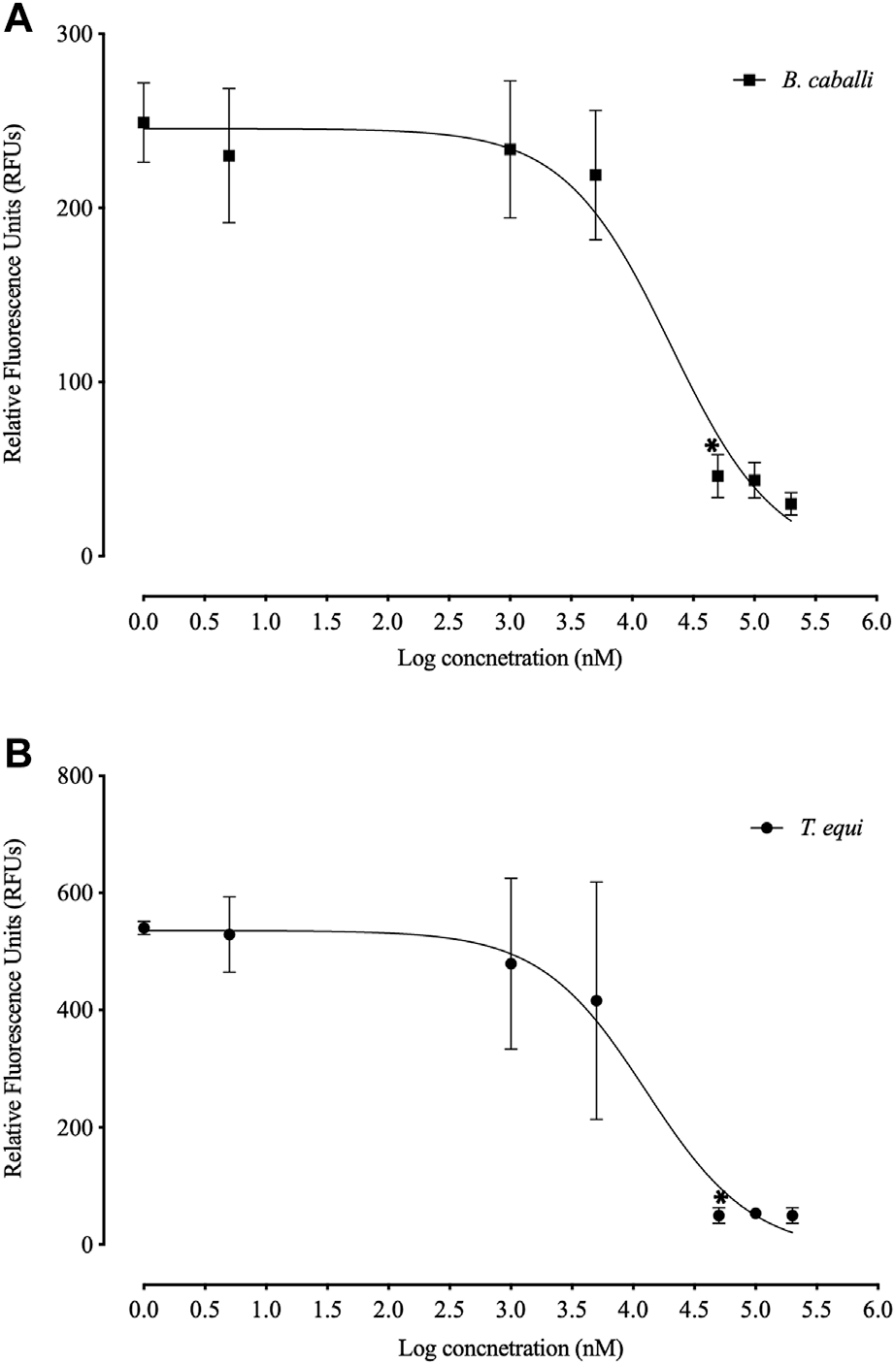
Correlation between relative fluorescence units (RFUs) and the log concentrations of FLLL-32 (µm) on equine piroplasmids. **(A)** *T. equi*. **(B)** *B. caballi*. Each value represents the mean of triplicate wells after subtraction of the background fluorescence for non-parasitized RBCs. The logarithmic values of drug concentration are of the base 10.

FLLL-32 treatments of 0.005-, 5, and 50 μM, respectively, significantly inhibited (*p* < 0.05) the *in vitro* growth of *B. bovis*, *B. bigemina*, and *B. divergens* (Figure 1). Meanwhile, 50, and 5 μM FLLL-32 treatments significantly inhibited (*p* < 0.05) the growth of *B. caballi*, and *T. Equi*, respectively (Figure 2). FLLL-32 exhibited the highest inhibitory effects on *B. bovis* growth, with an IC_50_ value of 9.57 ± 1.18 µM (Table 2). The estimated IC_50_ for *B. divergens*, *B. bigemina*, *B. caballi*, and *T. equi* were 26.46 ± 3.67, 28.14 ± 4.38, 28.95 ± 3.13, and 30.42 ± 1.54 µM, respectively (Table 1). After that, the regrowth of the parasites was assessed after stopping the treatment using the viability test. The results demonstrated that all tested parasites did not regrow at a dosage of ≥50 μM FLLL-32 (Supplementary Table S2). These findings suggest that FLLL-32 inhibits *B. bovis* growth more effectively than other bovine and equine piroplasmids *in vitro*.

**TABLE 2:** IC_50_ values of FLLL32 and diminazene aceturate evaluated for bovine *Babesia* and equine *Babesia* and *Theileria* parasites.

Organism		IC_50_(µM)^a^
	FLLL32	Diminazene aceturate^b^
*B. bovis*	9.57 ± 1.18	0.69 ± 0.07
*B. bigemina*	28.14 ± 4.38	1.31 ± 0.005
*B. divergens*	26.46 ± 3.67	0.38 ± 0.06
*T. equi*	30.42 ± 1.54	0.87 ± 0.04
*B. caballi*	28.95 ± 3.13	0.17 ± 0.006

^a^ IC_50_ values for FLLL32 and diminazene aceturate were calculated on the fourth day based on the growth inhibitions in three separate experiments. Each drug concentration was made in triplicate in each experiment, and the final obtained IC_50_ represents the mean and standard deviation of three separate experiments.

^b^ Traditionally used antibabesial drug (control drug).

To evaluate whether the *in vitro* inhibitory efficacy of FLLL-32 will increase when used in combination therapy, a combination consisting of FLLL-32 with either DA, ID, or MMV396693 was used. FLLL32 at a concentration of 0.75 x IC_50_ in combination with 0.5 x IC_50_ and 0.75 x IC_50_ DA showed additive effects against *B. bovis* on the fourth day of treatment (Table 3). FLLL-32 and MMV396693 combination at a concentration of 0.75 x IC_50_ exhibited an additive effect on *B. bigemina* (Table 4), meanwhile, the same combination showed synergistic interaction on the growth of *B. Caballi* (Table 5). FLLL-32 and ID combination at a concentration of 0.75xIC_50_ showed an additive effect on *B. caballi* (Table 5). Similarly, this drug combination (0.75 x IC_50_ FLLL32 and 0.75 x IC_50_ ID) showed synergistic interaction on the growth of *T. equi* (Table 6).

**TABLE 3:** Drug interactions of FLLL-32 in combination with diminazene aceturate (DA), imidocarb dipropionate (ID), and MMV396693 (MMV) on *B. bovis*.

Drug combination	M^a^	FIC_D1_	FIC_D2_	ΣFIC	Degree of interaction^b^
FLLL-32 _D1_ + DA_D2_	M1	0.26	0.32	0.58	Additive
	M2	0.31	0.22	0.54	Additive
	M3	0.77	0.52	1.29	Indifference
	M4	1.19	0.73	1.92	Indifference
FLLL-32 _D1_ + ID _D2_	M1	1.18	1.43	2.62	Antagonism
	M2	0.85	0.65	1.50	Indifference
	M3	1.44	0.75	2.19	Antagonism
	M4	1.61	0.94	2.55	Antagonism
FLLL-32 _D1_ + MMV _D2_	M1	0.80	0.38	1.18	Indifference
	M2	1.64	0.54	2.18	Antagonism
	M3	1.48	0.58	2.06	Antagonism
	M4	1.89	0.92	2.81	Antagonism

^a^ M1–4 refer to the combinations of FLLL-32, combined with different antibabesial drugs.

^b^ The degree of drug interaction was determined based on the following fractional inhibitory concentration (FIC) index: >0.5–1 (additive), >1 to <2 (indifferent), and ≥2 (antagonistic). FIC_D1_ refers to the fractional inhibitory concentration of FLLL-32. FIC_D2_ refers to the fractional inhibitory concentration of DA, ID, or MMV.

**TABLE 4:** Drug interactions of FLLL-32 in combination with diminazene aceturate (DA), imidocarb dipropionate (ID), and MMV396693 (MMV) on *B. bigemina*.

Drug combination	M^a^	FIC_D1_	FIC_D2_	ΣFIC	Degree of interaction^b^
FLLL-32 _D1_ + DA_D2_	M1	0.87	0.84	1.71	Indifference
	M2	1.63	0.85	2.48	Antagonism
	M3	2.50	0.73	3.23	Antagonism
	M4	1.01	1.00	2.01	Antagonism
FLLL-32 _D1_ + ID _D2_	M1	0.87	0.98	1.85	Indifference
	M2	1.00	1.00	1.99	Indifference
	M3	1.96	0.57	2.53	Antagonism
	M4	1.83	0.86	2.69	Antagonism
FLLL-32 _D1_ + MMV _D2_	M1	0.26	0.46	0.71	Additive
	M2	0.05	1.17	1.23	Indifference
	M3	0.63	2.07	2.70	Antagonism
	M4	0.97	1.33	2.29	Antagonism

^a^ M1–4 refer to the combinations of FLLL-32, combined with different antibabesial drugs.

^b^ The degree of drug interaction was determined based on the following fractional inhibitory concentration (FIC) index: >0.5–1 (additive), >1 to <2 (indifferent), and ≥2 (antagonistic). FIC_D1_ refers to the fractional inhibitory concentration of FLLL-32. FIC_D2_ refers to the fractional inhibitory concentration of DA, ID, or MMV.

**TABLE 5:** Drug interactions of FLLL-32 in combination with diminazene aceturate (DA), imidocarb dipropionate (ID), and MMV396693 (MMV) on *B. caballi*.

Drug combination	M^a^	FIC_D1_	FIC_D2_	ΣFIC	Degree of interaction^b^
FLLL-32 _D1_ + DA_D2_	M1	3.94	0.98	4.92	Antagonism
	M2	9.23	0.99	10.23	Antagonism
	M3	0.93	1.14	2.08	Antagonism
	M4	1.00	1.10	2.10	Antagonism
FLLL-32 _D1_ + ID _D2_	M1	0.57	0.15	0.73	Additive
	M2	4.46	0.82	5.28	Antagonism
	M3	0.65	1.76	2.41	Antagonism
	M4	0.98	1.94	2.92	Antagonism
FLLL-32 _D1_ + MMV _D2_	M1	0.16	0.01	0.18	Synergism
	M2	0.60	0.52	1.12	Indifference
	M3	0.84	0.87	1.71	Indifference
	M4	0.97	1.03	2.00	Indifference

^a^ M1–4 refer to the combinations of FLLL-32, combined with different antibabesial drugs.

^b^ The degree of drug interaction was determined based on the following fractional inhibitory concentration (FIC) index: ≤0.5 (synergetic), >0.5–1 (additive), >1 to <2 (indifferent), and ≥2 (antagonistic). FIC_D1_ refers to the fractional inhibitory concentration of FLLL-32. FIC_D2_ refers to the fractional inhibitory concentration of DA, ID, or MMV.

**TABLE 6:** Drug interactions of FLLL-32 in combination with diminazene aceturate (DA), imidocarb dipropionate (ID), and MMV396693 (MMV) on *T. equi*.

Drug combination	M^a^	FIC_D1_	FIC_D2_	ΣFIC	Degree of interaction^b^
FLLL-32 _D1_ + DA_D2_	M1	0.90	0.96	1.86	Indifference
	M2	1.41	0.93	2.34	Antagonism
	M3	1.92	0.89	2.81	Antagonism
	M4	0.99	1.04	2.03	Antagonism
FLLL-32 _D1_ + ID _D2_	M1	0.80	0.84	1.64	Indifference
	M2	1.45	0.92	2.37	Antagonism
	M3	1.82	0.82	2.64	Antagonism
	M4	1.00	1.02	2.02	Antagonism
FLLL-32 _D1_ + MMV _D2_	M1	0.02	0.42	0.45	Synergism
	M2	0.40	0.86	1.26	Indifference
	M3	0.61	1.11	1.73	Indifference
	M4	0.88	0.88	1.76	Indifference

^a^ M1–4 refer to the combinations of FLLL-32, combined with different antibabesial drugs.

^b^ The degree of drug interaction was determined based on the following fractional inhibitory concentration (FIC) index: ≤0.5 (synergetic), >0.5–1 (additive), >1 to <2 (indifferent), and ≥2 (antagonistic). FIC_D1_ refers to the fractional inhibitory concentration of FLLL-32. FIC_D2_ refers to the fractional inhibitory concentration of DA, ID, or MMV.

### 3.2 FLLL-32 inhibited the mRNA transcription of *BbSAHH*

**FIGURE 3:**
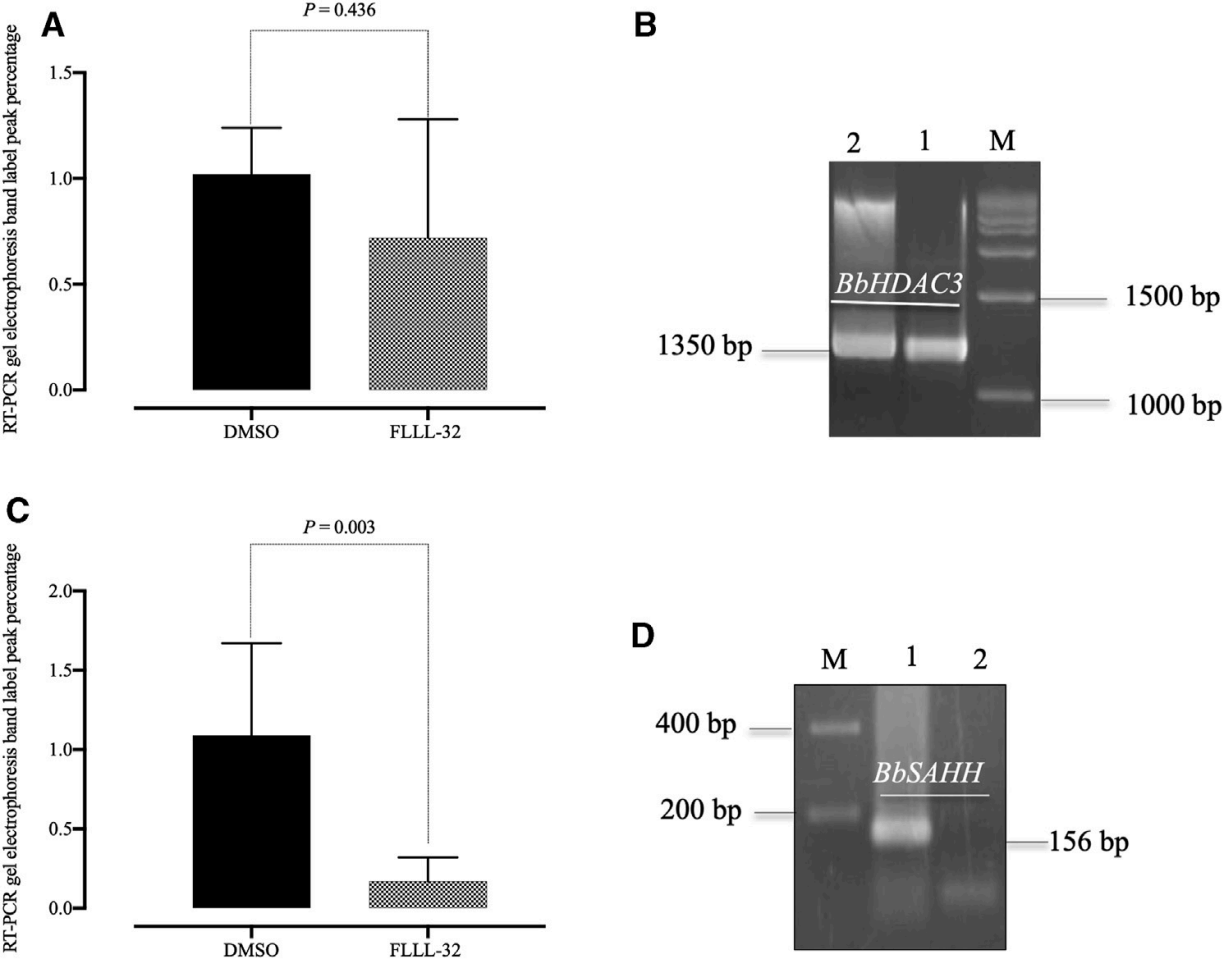
Reverse transcription-PCR analysis of *B. bovis HDAC3* and *B. bovis SAHH* genes from *B. bovis* cultures treated with FLLL32 at IC_99_ concentration and DMSO (0.1%) used as a control for 8 h. **(A)** Percentages of gel electrophoresis band peak for *B. bovis HDAC3*. **(B)** Gel electrophoresis for *B. bovis HDAC3* (lane 1, FLLL32 -treated culture; lane 2, control culture). **(C)** Percentages of gel electrophoresis band peak for **(B)** *B. bovis SAHH.* **(D)** Gel electrophoresis for *B. bovis SAHH* (lane 1, control culture; lane 2, FLLL32 -treated culture). M, molecular size marker. The percentages were determined by ImageJ software.

In the *B. bovis* culture, at the IC_99_ concentration, the FLLL-32 doesn’t affect the transcription of the Bb*HDAC3* gene in 8 h treatments as it was shown by comparison of the level of mRNA transcript of this gene in treated cells with those of controls (cells treated with 0.1% DMSO) (Figure 3). Interestingly, in these treatments, the expression of the other *B. bovis* gene, Bb*SAHH* in the cells treated by FLLL-32 was completely inhibited while its level in controls was not changed (Figure 3). The expression level of Bb*PROF* gene did not differ between treated cells and controls (data not shown).

### 3.3 FLLL-32 inhibits *B. microti* in mice

**FIGURE 4:**
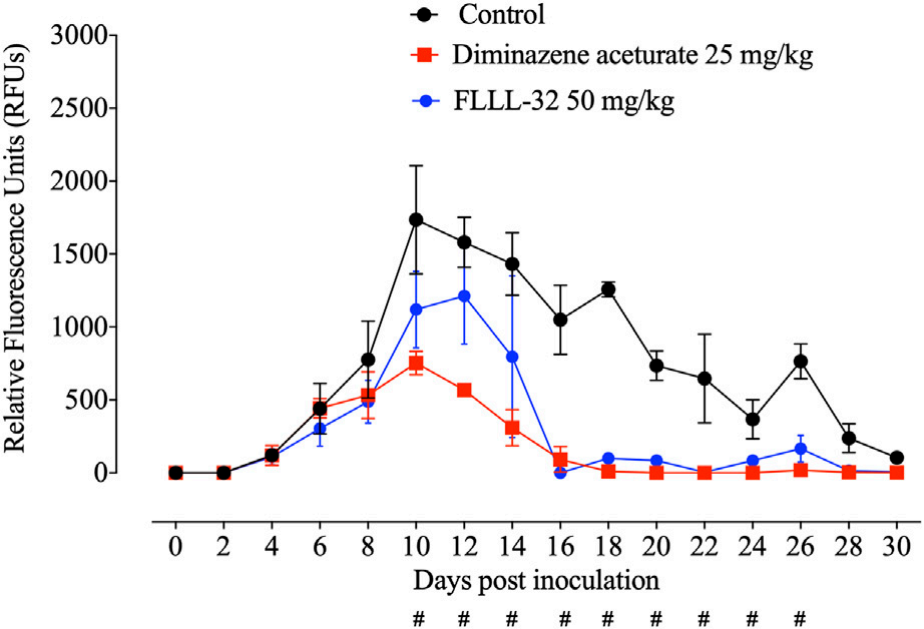
Inhibitory effect of FLLL-32 on the growth of *Babesia microti*. Each value represents the mean ± standard deviation of five mice per experimental group. Diminazene aceturate (DA) was used as the control drug and the non-treated group was used as negative control mice. Asymbol indicates significant differences (#*p* < 0.05) from day 10 to day 26 post-inoculation between the FLLL-32–treated and control groups.

Parasitemia levels were significantly reduced (*p* < 0.05) in mice treated with FLLL-32 from day10 post infection (pi) to reach zero level at day 16 pi in comparison with the non-treated control group (Figure 4). Treatment with 50 mg/kg FLLL-32 resulted in 35% inhibition at day 10 p.i. (peak of parasitemia) (Figure 4). Peak fluorescence values in the treated groups with FLLL-32 50 mg/kg reached an average of 1213 at day 12 pi. Fluorescence readings were significantly reduced (*p* < 0.05) in mice treated with FLLL-32 from days 10–26 p.i. When compared to positive control mice (infected nontreated) which is similar to DA-treated mice (Figure 4). The obtained results suggested the hopeful antibabesial efficacy of FLLL-32 in an infected experimental animal model.

## 4 Discussion

Emerging data suggest that natural products may represent effective candidate molecules for drug discovery, however, their clinical utility is somewhat limited due to the poor bioavailability and target selectivity (Ćavar Zeljković et al., 2022). Therefore, efforts are underway to design and synthesize novel analogs with a higher bioavailability and target specificity (Bill et al., 2010a; Ćavar Zeljković et al., 2022). FLLL-32 is one of the curcumin analogs which modeled based on the diketone form of curcumin binding to the SH2 domain of STAT3 (Yang and Lesinski, 2012). Curcuma and its associated bioactive compounds showed some antiprotozoal activities against *Plasmodium*, *Leishmania*, *Trypanosoma*, *Babesia*, and *Giardia* (Haddad et al., 2011). These derivatives are shown to have several pharmacological effects such as anti-inflammatory, antioxidant, anticarcinogenic, antibacterial, antifungal, antiprotozoal, antiviral, and immunomodulatory (Abd El-Hack et al., 2021).

General talking, *B. bovis*, *B. bigemina*, and *B. divergens* are the most economically important bovine babesiosis (Rizk et al., 2020). However, *B. bovis* infection is associated with more severe disease and higher mortality and is considered the most virulent species of bovine babesiosis (Schnittger et al., 2012; Gohil et al., 2013; Suarez et al., 2019). Because of the limitations of chemoprophylaxis and acaricide control of transmitting vectors, live attenuated vaccine immunization against *B. bovis* has been used as an alternate control method (Rizk et al., 2020). However, several drawbacks associated with the production of these control methods have been identified (Cuy-Chaparro et al., 2023). Therefore, safer anti-babesial medications that either cure the infection or reduce the dosages of DA, and ID supplied are likely to be more effective methods.

In the present study, FLLL-32 showed potent *in vitro* inhibitory effects against the growth of various *Babesia* species and exhibited the highest inhibitory effects on *B. bovis* growth. Interestingly, the IC_50_ of FLLL-32 for *B. bovis* was lower than those previously obtained with other antibabesial drugs including gedunin (17.86 µM) (Rizk et al., 2015), N-acetyl-l-cysteine (332.11 µM) (Rizk et al., 2017), enoxacin (38.04 µM) (Rizk et al., 2018), and thymoquinone (35.41 µM) (El-Sayed et al., 2019; Rizk et al., 2020). In general, several factors related to the screening parasite, including parasite type, strain, and size, affect the effectiveness of the tested medications (Rizk et al., 2019; Rizk et al., 2020). The medium used, the HCT, and whether or not serum is present in the *in vitro* culture have an impact on the calculated IC_50_s of the medicine being tested (Rizk et al., 2015; Rizk et al., 2016). As a result, the inconsistent FLLL-32 IC_50_ values in the current investigation could be explained by variations in the parasite species or culture conditions between the several screened piroplasm.

Indeed, several antibabesial compounds obtained from natural herbal sources were evaluated against babesiosis in our laboratory. For example, allicin and fusidic acid (Salama et al., 2013; Salama et al., 2014) exhibited much higher IC_50_ values (µM) against *B. bovis*, *B. bigemina*, *B. caballi*, and *T. equi* than those calculated for FLLL-32. In the same way, turmeric methanolic extract showed *in vitro* inhibitory activity against *B. divergens*, *B. caballi*, and *B. bovis* higher than those estimated for FLLL-32 (Rizk et al., 2021b). Of note, the *in vivo* inhibition of FLLL-32 against *B. microti* in mice at day with peak parasitemia (day 10) was higher than those caused by 100 mg/kg enoxacin (21%), 150 mg/kg norfloxacin (15%), and 700 mg/kg ofloxacin (23%) (Rizk et al., 2018).

FLLL32 has exceptional biochemical properties, and in particular inhibits signal transducer and activator of transcription 3 (STAT3) phosphorylation, DNA-binding activity, and transactivation, and demonstrates significant growth suppressive activity in a variety of human cancer cells (Lin et al., 2010a; Bill et al., 2010b; Lin et al., 2010b; Fossey et al., 2011; Wei et al., 2011; Bill et al., 2012). Furthermore, FLLL32 can suppress IFNα and interleukin-6-induced STAT3 phosphorylation (Lin et al., 2010a; Onimoe et al., 2012). Curcuminoids have been identified as JMJD2 histone demethylase inhibitors, with FLLL-32 inhibiting only JMJD2D (Kim et al., 2014). It has been proposed that FLLL-32 reduces intestinal damage in necrotizing enterocolitis (Eckert et al., 2017). In this study, we investigated the effects of FLLL-32 treatment on two *B. bovis* genes Bb*HDAC3* and Bb*SAHH*. However, the effect of apicidin which is an inhibitor of histone deacetylase (HDAC) on *Babesia* parasite growth has been previously reported (Munkhjargal et al., 2009; Munkhjargal et al., 2012) but FLLL-32 showed no effects on the mRNA transcription. On the contrary, the mRNA transcription of the Bb*SAHH* of cultures treated with FLLL-32 was significantly inhibited (*p* < 0.05) but not the mRNA transcription of the control BbPROF gene in comparison to cultures treated with DMSO (0.1%) within 8 h of treatment.

The enzyme S-adenosylhomocysteine hydrolase (SAHH) catalyzes the reversible breakdown of S-adenosylhomocysteine (SAH) to homocysteine and adenosine (De La Haba, and Cantoni, 1959). Homocysteine and adenosine product elimination are necessary for SAH hydrolysis. SAH accumulation can inhibit methyltransferase activity by having a high affinity binding to the enzyme active site in the absence of effective product elimination (Hu et al., 1999; Yi et al., 2000). Because it can alter the cellular methylation of phospholipids, proteins, small molecules, DNA, and RNA, SAHH has become an attractive pharmaceutical target (Chiang, 1998). Several nucleoside inhibitors of SAHH have therefore been synthesized, having pharmacological and biological effects such as antiviral effects (Chiang, 1998; Clerq, 1998). S-adenosylhomocysteine hydrolase was shown to be an interesting target for the development of novel anti-malarial agents (Bitonti et al., 1990; Kitade et al., 2003; Nakanishi et al., 2005; Chandra et al., 2021). SAHH gene from *Plasmodium falciparum* (PfSAHH) was well characterized (Creedon et al., 1994; Bujnicki et al., 2003; Tanaka et al., 2004) however, no studies have been conducted on *B. bovis*. High binding affinity with PfSAHH has been found for curcumin and its derivatives which curcumin as a potential candidate for the development of antimalarial drugs (Singh et al., 2013). Altogether, SAH hydrolase may be a promising pharmacological target for developing antibabesial drugs, particularly for the most virulent species, *B. bovis*. Although, the present study evaluated the *in vitro* and *in vivo* antibabesial efficacy of FLLL-32, future studies are required to assess the effect of this drug on the developmental growth arrest using the phenotypic assay.

## 5 Conclusion

The curcumin analog FLLL-32 showed potent inhibitory effects on the *in vitro* growth of *B. bovis*, *B. bigemina*, *B. divergens*, *B. caballi*, and *T. equi* with *B. bovis* being the most susceptible species. FLLL-32 was shown to inhibit the enzyme S-adenosylhomocysteine hydrolase (SAHH) of *B. bovis* that can cause increasing the SAH to S-adenosylmethionine (SAM) ratio and blocking SAM-dependent methyltransferase, which catalyzes the methylation process required for parasite growth. Moreover, the compound showed antibabesial activities against the *in vivo* growth of *B. microti* in infected mice. Further studies are required to characterize the SAHH enzyme of *B. bovis* and to investigate the inhibitor’s interaction especially FLLL-32 with this drug target.

## Data Availability

The raw data supporting the conclusion of this article will be made available by the authors, without undue reservation.
