# Supplementary material for: Antiparasitic activity of FLLL-32 against four Babesia species, B. bovis, B. bigemina, B. divergens and B. caballi, and one Theileria species, Theileria equi in vitro, and Babesia microti in mice
Source: Front Pharmacol. 2023 Nov 2;14:1278451. doi: 10.3389/fphar.2023.1278451 (PMC10651744; doi:10.3389/fphar.2023.1278451)
Supplement: Supplementary file 1 [file DataSheet1.PDF]

Table S1. Concentrations of FLLL32 combined with diminazene aceturate (DA), imidocarb dipropionate (ID), and MMV396693 (MMV) applied to the cultures of bovine *Babesia* and equine *Babesia* and *Theileria* parasites

M[a]	IC_{50} [b]			
	**FLLL32**	**DA**	**ID**	**MMV**
**M1**	¾	¾	¾	¾
**M2**	¾	½	½	½
**M3**	½	¾	¾	¾
**M4**	½	½	½	½

[a] M1- 4 refers to the combinations of FLLL-32 combined with different antibabesial drugs . [b] Combinations were based on the calculated IC_{50} values obtained from the *in vitro* fluorescence- based assay

Table S2. Viability test results of FLLL-32 drug evaluated for *Babesia* and *Theileria* parasite

Drug	Drug concentrations (µM) [a]					
	0.05	1	5	50	100	200
B. bovis	+	+	+	−	−	−
B. bigemina	+	+	+	−	−	−
B. divergens	+	+	+	−	−	−
T. equi	+	+	+	−	−	−
B. caballi	+	+	+	−	−	−

[a] Each value was calculated in three separate experiments. Each concentration of the drug was made in triplicate in each experiment. + = viable; − = dead
